# Association of Immunosuppression with DR6 Expression during the Development and Progression of Spontaneous Ovarian Cancer in Laying Hen Model

**DOI:** 10.1155/2016/6729379

**Published:** 2016-08-04

**Authors:** Sa'Rah McNeal, Pincas Bitterman, Janice M. Bahr, Seby L. Edassery, Jacques S. Abramowicz, Sanjib Basu, Animesh Barua

**Affiliations:** ^1^Rush University Medical Center, Chicago, IL 60612, USA; ^2^University of Illinois at Urbana-Champaign, Urbana, IL 61801, USA; ^3^University of Chicago, Chicago, IL 60637, USA

## Abstract

Ovarian cancer (OVCA) mainly disseminates in the peritoneal cavity. Immune functions are important to prevent OVCA progression and recurrence. The mechanism of immunosuppression, a hallmark of tumor progression, is not well understood. The goal of this study was to determine the immune system's responses and its suppression during OVCA development and progression in hens. Frequencies of CD8+ T cells and IgY-containing cells and expression of immunosuppressors including IRG1 and DR6 in OVCA at early and late stages in hens were examined. Frequencies of stromal but not the intratumoral CD+8 T cells and IgY-containing cells increased significantly (*P* < 0.01) during OVCA development and progression. Tumor progression was associated with increased expression of IRG1 and DR6 and decreased infiltration of immune cells into the tumor. Frequency of stromal but not intratumoral immune cells increases during OVCA development and progression. Tumor-induced IRG1 and DR6 may prevent immune cells from invading the tumor.

## 1. Introduction

Ovarian cancer (OVCA) is a fatal malignancy in women with high case-to-death ratio [1]. The rate of survival of OVCA patients is remarkably high when it is detected at an early stage as compared with late stages [2, 3]. Due to the nonspecificity of symptoms at early stage and the lack of an early detection test, OVCA in most cases is detected at late stages with a 5-year survival rate of patients of <20% as opposed to >90% when detected at an early stage [3]. Moreover, development of resistance to currently available chemotherapeutics and frequent recurrence of OVCA contribute to the poor prognosis of the disease. Thus, aggressive growth rates together with ineffective conventional chemotherapeutics and subsequent recurrence are associated with high rates of death of OVCA patients. Therefore, information on factors associated with tumor survival, progression (metastasis), and recurrence is critical for designing the interventional strategies to prevent the high rate of death associated with OVCA.

During the process of malignant transformation, developing tumors express neoantigens. These neoantigens are considered as “foreign” by the body's immune system; as a result, it elicits immune responses against a growing tumor. However, spontaneous rejection of an established tumor is rare, suggesting inefficacy of the immune system in eradicating a tumor and/or existence of a condition for suppression of antitumor immune function [4]. Ovarian tumors, in contrast to other solid tumors, progress mainly through local diffusion, in addition to systemic dissemination, and immune cells in the tumor microenvironment play critical roles in preventing tumor progression or metastasis. However, as the tumor continues to grow, it may induce factors required to suppress antitumor immunity in the local tumor microenvironment. Immunosuppression induced by ovarian tumors is poorly understood. Thus, information on tumor-induced factors associated with immunosuppression is essential for defining the interventional strategies required for the prevention of tumor metastasis and recurrence. Emerging information suggests that death receptor 6 (DR6) is an immunosuppressive factor shown to be secreted by malignant cells in several cancers. Death receptor 6 is a member of the tumor necrosis factor *α* receptor superfamily 21 (TNFRSF21) [5, 6].

In contrast to other members of TNFRSF, increased tissue expression of DR6 has been reported in association with the progression of cancers of several organs [7]. An increased expression of DR6 was reported to be associated with the progression of prostate cancer [7]. On the other hand, increase in DR6 gene expression was not observed in lung and colorectal cancer cell lines. Thus, changes in DR6 expression may vary in relation to the sites of malignancies. Information on the changes in DR6 expression by ovarian malignant cells in patients during OVCA development, progression, and recurrence is not known. Monocytes are an important member of the innate immune system, and DR6 has been suggested to stimulate switching normal differentiation of monocytes into immature dendritic cells (iDCs) in lieu of mature dendritic cells (mDCs) [8]. Immature dendritic cells are involved in the induction of tolerance [9]. Furthermore, DR6 has also been suggested to be immunosuppressive as it is involved in the inhibition of proliferation and migration of T and B cells. Thus, it is possible that expression of DR6 by ovarian malignant cells may be involved in the induction of immunosuppression aiding tumor survival, progression, and recurrence [10].

Increase in serum levels of DR6 was associated with late stage OVCA patients [11, 12]; however, changes in DR6 expression during ovarian malignant transformation and early stage OVCA are not known. Difficulty in identification of patients at early stage and access to them is a significant barrier to study the association of DR6 with OVCA-induced immunosuppression. Rodents do not develop OVCA spontaneously and induced OVCA differs from spontaneous OVCA both in histological types and in the mode of dissemination. In contrast, laying hens develop OVCA spontaneously with similarities in histopathology together with high rates of incidence [13, 14]. Similarities between hens and humans also exist in reproductive physiology including endocrine regulation of ovarian functions. Moreover, laying hens are also a model of the consequence of incessant ovulation and OVCA development [15]. In addition, OVCA in laying hens has been shown to express several markers including DR6 similarly expressed in human OVCA [16–18]. Furthermore, laying hens have contributed immensely to our understanding of the immune system [19]. Thus, the goal of this exploratory study was to examine (1) whether cell mediated cytotoxic and humoral immune function in the ovary changes in response to spontaneous ovarian tumor development and progression and (2) whether ovarian tumor progression is associated with tumor-induced suppression of antitumor immune function.

## 2. Materials and Methods

### 2.1. Animals

Four-year-old commercial strains of White Leghorn laying hens (*n* = 120) reared under standard husbandry practices were selected based on their status of egg laying including cessation or low rates of egg laying as reported previously [20]. Rate of incidence of spontaneous OVCA in hens of this age group is ~20% and is accompanied with low or complete cessation of egg laying [21, 22]. All husbandry and experimental procedures were performed according to the institutional animal care and use committee (IACUC) approved protocol.

### 2.2. Collection and Processing of Tissues

#### 2.2.1. Serum Samples

Blood samples were collected from wing veins (brachial veins) of all hens before sacrifice and centrifuged (1,000 ×g, 10 min). Sera were separated and aliquots were stored at −80°C for later use.

#### 2.2.2. Ovarian Tissue Sample

Normal ovarian tissues or ovaries with tumors were collected following euthanasia. Gross examination of hens was performed for abnormal presentation of various organs including ovaries and oviducts. Presence of ovarian tumors and their stages were classified based on the extent of dissemination of the disease including the presence or absence of ascites as reported earlier [13]. When solid mass was present both in the ovaries and in the oviducts, inner layers of oviduct tissues were examined to determine the presence of any solid mass. Hens with solid mass appearing in the inner side of the oviducts were discarded from the study. Tissues from normal ovaries or malignant ovaries were divided into four portions for paraffin and frozen sections as well as protein and total RNA collection. Paraffin embedded tissues were used for routine histology to determine the histological types of ovarian cancer while both paraffin and frozen sections were used for immunohistochemical studies as reported previously [22]. In addition to tumor tissues, normal ovarian surface epithelial (OSE) cells or ovarian malignant epithelial cells were collected as reported earlier [23, 24]. Collected samples were classified into 3 groups including normal, early stage OVCA, and late stage OVCA based on gross examinations and routine microscopy as previously reported [13].

### 2.3. Processing of Ovarian Samples for Proteomic Studies

Collected snap frozen tissues (mentioned above) were homogenized using a Polytron homogenizer (Brinkman Instruments, Westbury, NY) as reported previously [21] and centrifuged. Following centrifugation, supernatants were collected; their protein contents were measured and stored at −80°C for later use.

### 2.4. Immunohistochemistry

Rabbit polyclonal anti-DR6, anti-CD8, anti-IgY, and anti-IRG1 (immune responsive gene 1 protein) antibodies were used as primary antibodies and immunoreactions were determined using Vectastain Elite ABC kit (Universal, RTU, Vector Lab Inc., Burlingame, CA). Normal or malignant ovaries [normal, early, and late stages] were used for immunohistochemical study as determined by the power analysis to achieve significant differences in different parameters among hens of different pathological groups. For determination of CD8 T cells and IRG1 expression, frozen sections were used as reported earlier [25, 26]. Control staining was carried out simultaneously in which the first antibodies were omitted and normal serum was used. No staining was found in these control slides. Sections were then examined under a light microscope attached to digital imaging software (MicroSuite version 5; Olympus Corporation, Tokyo, Japan). The intensities of IRG1 and DR6 staining as well as the frequencies of CD8+ T cells and IgY-expressing cells in different groups including normal and OVCA hens were determined. Three sections per ovary and 5 regions of interest with the highest immunoreactivity (ROI, 20,000 *μ*m^2^/region at ×40 objective and ×10 ocular magnification) per section were selected. Using the software, the intensity of the IRG1 and DR6 immunostaining in each region was measured and recorded as arbitrary values in 20,000 *μ*m^2^of the tissue as reported previously [27]. The mean of arbitrary values of these 5 regions in a section was considered as the intensity of IRG1 and DR6 in 20,000 *μ*m^2^area of tissue. The mean intensity of 3 sections was considered as the IRG1 and DR6 staining intensity in 20,000 *μ*m^2^ area of each normal or malignant ovary. The group-wise IRG1 and DR6 staining intensity (normal or tumor groups) was expressed as mean ± SEM in 20,000 *μ*m^2^ area of tissue in normal or malignant groups. Similarly, using the same software, the frequencies of CD8+ T cells and IgY-expressing cells in the sections were counted and reported as the average frequency (mean ± SEM) cells in 20,000 *μ*m^2^ area of the tissue as reported previously [27–29].

### 2.5. One-Dimensional (1D) Western Blot

Ovarian expression of IRG1 and DR6 was confirmed by immunoblotting using homogenates of normal (*n* = 5) or malignant ovaries at early and late stages. Representative samples of serous, endometrioid, and mucinous carcinoma at early and late stages of OVCA were selected for immunoblotting based on their immunoreactivity for IRG1 and DR6 in immunohistochemistry. *β*-actin was used as control for determining changes in protein expression relative to disease status. Immunoreactions on the membrane were visualized as a chemiluminescence product (Super Dura West substrate; Pierce) and the images were captured using ChemiDoc XRS (Bio-Rad) [21].

### 2.6. Reverse Transcription-Polymerase Chain Reaction (RT-PCR)

IRG1 and DR6 mRNA expression was assessed by semiquantitative RT-PCR as reported previously [30]. For RT-PCR analyses, representative tissue samples from normal, early stage, and late stages of OVCA hens were selected based on their reactivity in immunohistochemistry and immunoblotting. Chicken-specific DR6 primers were designed by OligoPerfect Designer software (Invitrogen, Carlsbad, CA) using the DR6 sequence from the NCBI (accession number A1980074) as reported earlier [18, 31]. The forward primer was 5′-GAT GGA GGA CAC CAC GCC-3′ and the reverse primer was 5′-TCG GGG TTG AGG ATG TGC-3′. *β*-actin was used as the endogenous control with a forward primer of 5′-TGCGTGACATCAAGGAGAAG-3′ and a reverse primer of 5′-ATGCCAGGGTACATTGTGGT-3′. The expected base pair size for the DR6 amplicon was 384 bp and for *β*-actin was 300 bp. PCR amplicons were visualized in a 3% agarose gel (Pierce/Thermo Fisher, Rockford, IL, USA) in TAE buffer and stained with ethidium bromide. The image was captured using a ChemiDoc XRS system (Bio-Rad, Hercules, CA). Similarly, for IRG1 mRNA expression, the forward primer was 5′-GAAGCGGCTTGCTTAGCATC-3′ and the reverse primer was 5′-TCCAACAGCCAGGGATAGGA-3′.

### 2.7. Statistical Analysis

The differences in the frequency of CD8+ T cells and IgY-expressing cells as well as in the intensity of IRG and DR6 immunostaining in normal ovaries and malignant ovaries were assessed by ANOVA, *F*-tests, and the alternative nonparametric Kruskal-Wallis tests. Subsequently, pairwise comparisons between the groups (normal, early, and late stage OVCA) by two-sample *t*-tests and alternative Mann-Whitney tests were performed. Statistical analyses were performed using GraphPad Prism software (GraphPad Software Inc., San Diego, CA). Data are presented as mean + SEM and *P* < 0.05 was considered as statistically significant.

## 3. Results

### 3.1. Ovarian Gross Morphology

**Figure 1:**
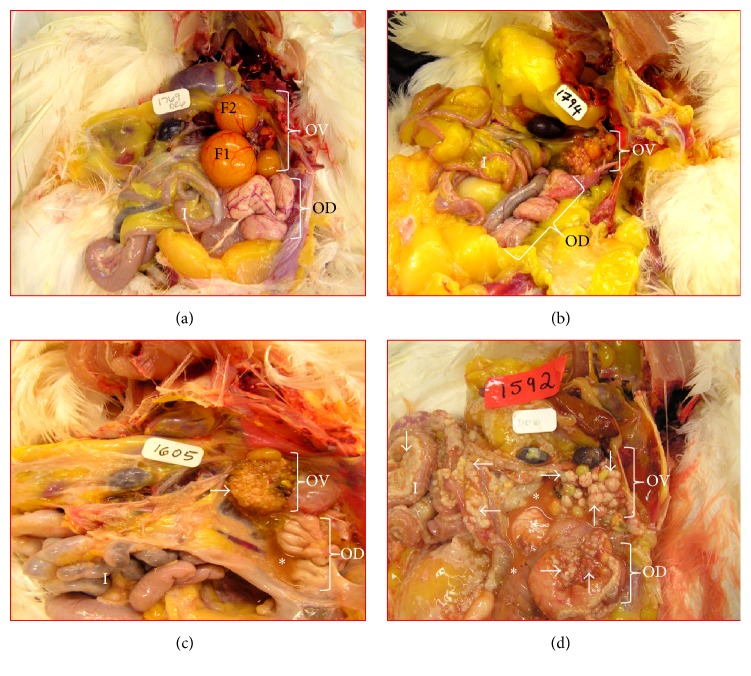
Gross presentation of spontaneous ovarian cancer (OVCA) in hens. (a) Ovary (OV) of a normal old laying hen showing few large preovulatory follicles (F1 and F2) and small developing follicles. The oviduct (OD) appears to be functional and no abnormalities are detected. (b) Ovary of a hen out of lay without large follicles. Both the ovary and the oviduct regressed remarkably. (c) Early stage OVCA in a hen. Tumor limited to the ovary with little ascites (*∗*). (d) Late stage OVCA in a hen accompanied with profuse ascites (*∗*). Tumor metastasized to other organs (arrows indicate examples of tumor metastasis).

In contrast to the 5-6 preovulatory follicles in a fully functional ovary of a 1-2-year-old young laying hen, the ovary in an apparently healthy 4-year-old hen with low egg laying rate contains 2-3 large preovulatory follicles (Figure 1(a)). On the other hand, the ovary in an apparently healthy hen that ceased or stopped laying becomes atrophied or regressed without any developing follicle resembling a postmenopausal ovary in a woman (Figure 1(b)). The atrophy of the ovary is accompanied with the atrophy of the oviduct. Of 120 hens, solid masses were observed in 22 hens. Tumors in 8 of these hens were limited either to a small area or to the entire ovary, with little or no accompanying ascites. These hens were diagnosed to have early stage OVCA (Figure 1(c)). In the remaining 14 hens, the tumor metastasized to distant organs accompanied with profuse ascites. These hens were diagnosed to have late stage OVCA (Figure 1(d)).

### 3.2. Microscopic Evaluation

**Figure 2:**
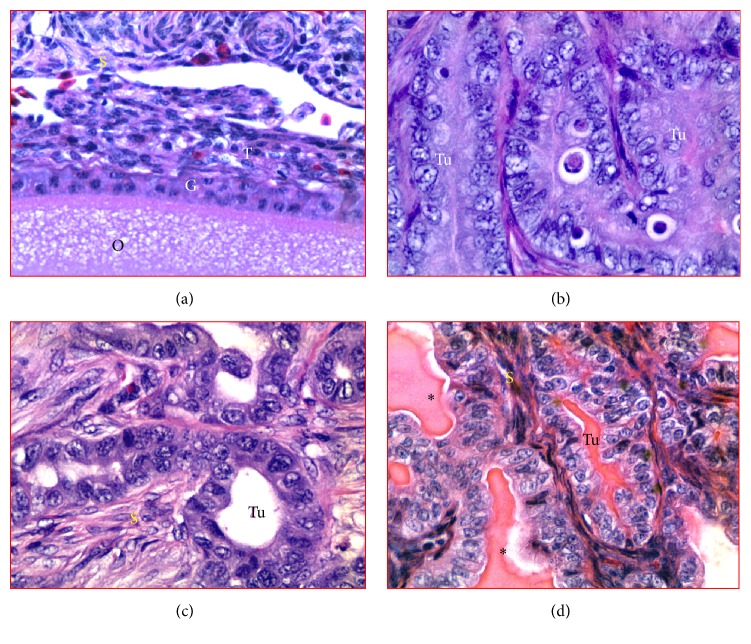
Observed histologic subtypes of spontaneous ovarian cancer in hens. (a) Section of normal ovary showing a cortical follicle embedded in the stroma. (b) Ovarian serous carcinoma showing a sheath-like structure containing cells with large pleomorphic nuclei and mitotic bodies. (c) Ovarian endometrioid carcinoma showing back-to-back confluent tumor (Tu) glands consisting of a single layer of cuboidal epithelial cells with sharp luminal margin. (d) Ovarian mucinous carcinoma showing the tumor with crowded glands containing very thin intervening stroma. The tumor contains a columnar layer of intercalated ciliated goblet cells with abundant mucin-like luminal secretion (*∗*). G and T: granulosa and theca layers of follicle (F); S: stroma; Tu: tumor. H&E, 40x.

In normal ovaries, ovarian stroma consists of embedded cortical follicles of various sizes containing developing granulosa cell layer and theca layers (Figure 2(a)). The medulla consists of large and smaller vascular tissues located at the center of the ovary. Histological presence of tumors was confirmed by microscopic examination in hens that had solid masses detected at gross examination following euthanasia (Figures 2(b)–2(d)). However, microscopic malignant features including large cells with pleomorphic nuclei arranged in focal lesions were also detected during histological examinations in 2 additional hens without any grossly detectable solid tumor mass and were considered as early stage OVCA. Overall, 10 (8 + 2) hens had early stage and 14 hens had late stage OVCA. No abnormality was detected in the remaining 96 hens. Following microscopic examinations, tumors were classified histopathologically as reported previously [13] and 10 hens had serous, 8 had endometrioid, and 4 had mucinous OVCA while 2 had mixed (1 seromucinous and 1 endomucinous) OVCA.

### 3.3. Enumeration of CD8+ T Cells and IgY-Containing Cells

**Figure 3:**
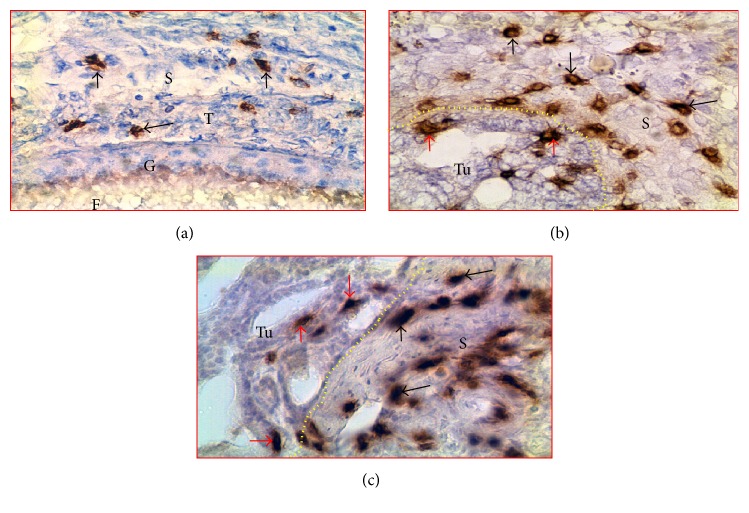
Localization of CD8+ T cells in hen ovaries with or without tumors. (a) Normal ovary showing CD8+ T cells. (b-c) Ovarian tumors at early and late stages showing more CD8+ T cells in tumor stroma. Few CD8+ T cells are seen to invade the tumor. Although influx of CD8+ T cells into the stroma increased with OVCA progression, intratumoral population of CD8+ T cells did not increase. Black and red arrows indicate examples of stromal and intratumoral CD8+ T cells, respectively. Dotted lines indicate the tumor. G and T: granulosa and theca of follicle (F); S: stroma; Tu: tumor; 40x.

In normal ovaries, immunopositive CD8+ T cells were localized in the ovarian stroma and theca of the stromal embedded follicle (Figure 3(a)). In the ovaries with tumors, CD8+ T cells were seen in the tumor stroma (spaces in between the tumor cores). Compared with the ovarian stroma in normal hens (Figure 3(a)), more CD8+ T cells were observed in the stroma of ovaries with tumors at early stage (Figure 3(b)) and late stage (Figure 3(c)) OVCA. In addition, few CD8+ T cells were also seen to be infiltrating into the tumor nests.

**Figure 4:**
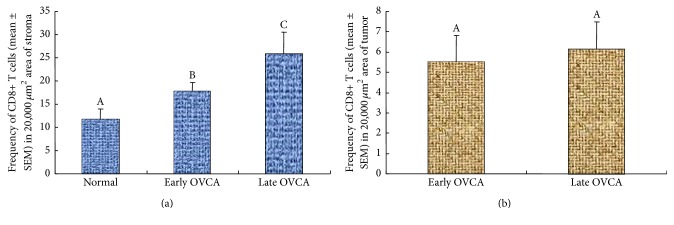
Changes in the frequency of CD8+ T cells in the ovaries of hens during the development and progression of ovarian cancer (OVCA). (a) Compared to the normal, frequency of CD8+ T cells was significantly greater (*P* < 0.01) in the stroma of the tumor at early stage and increased further in late stage OVCA. (b) In contrast to the influx of stromal CD8+ T cells, the frequency of the intratumoral CD8+ T cells did not increase significantly during OVCA progression to late stage. Bars with different letters indicate significant differences in CD8+ T cells among hens of different pathological groups.

The population of CD8+ T cells was counted separately by their localization as stromal (stroma of the normal ovaries or ovaries with tumor) and intratumoral including those infiltrating into the tumor nests. The mean frequency of CD8+ T cells in the stroma of normal hens was approximately 6.0 ± 1.30 (mean ± SEM) cells in 20,000 *μ*m^2^ of the tissue. Compared with normal hens, the mean frequency of CD8+ T cells was significantly greater (mean ± SEM = 18 ± 1.80 cells, *P* < 0.01) in the tumor stroma at early stage and increased further in the stroma of tumors at late stage OVCA (mean ± SEM = 26 ± 1.83 cells, *P* < 0.05) in 20,000 *μ*m^2^ of the tissue (Figure 4(a)). The mean frequency of intratumoral CD8+ T cells in hens with early stage OVCA was 5 ± 1.30 (mean ± SEM) cells in 20,000 *μ*m^2^ area of the tissue. Although, compared with hens with early stage OVCA, the frequency of stromal CD8+ T cells was higher in hens with late stage OVCA, the mean frequency of intratumoral CD8+ T cells in late stage OVCA (mean ± SEM = 6 ± 1.35 cells in 20,000 *μ*m^2^ area of the tissue) did not increase significantly (*P* < 0.4) (Figure 4(b)).

**Figure 5:**
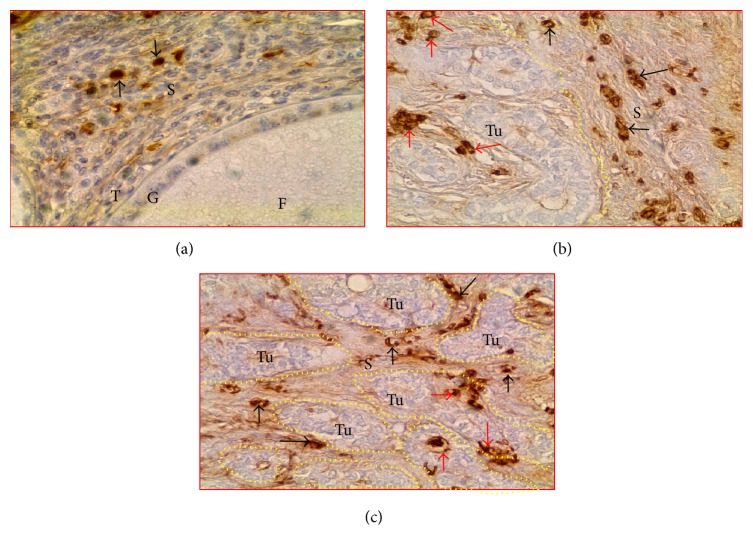
Immunodetection of IgY-containing cells in normal and tumor ovaries in hens. (a) Few IgY-containing cells are seen in the normal ovary. (b-c) Ovarian tumors at early and late stages. More IgY-containing cells are seen in the tumor stroma. Only a few IgY-containing cells are seen to be infiltrating into the tumor. Although influx of IgY-containing cells into the tumor stroma increased with OVCA progression, intratumoral population of IgY-containing cells did not increase. Black and red arrows indicate the examples of stromal and intratumoral IgY-containing cells, respectively. Dotted lines indicate the tumor in the stroma. Other legends are similar to Figure 3; 40x.

**Figure 6:**
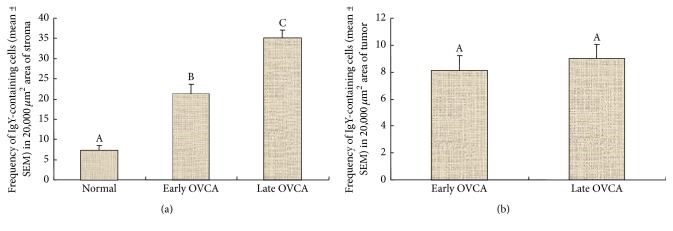
Changes in the frequency of IgY-containing cells in the ovaries of hens during OVCA development and progression. (a) The population of stromal IgY-containing cells was significantly greater (*P* < 0.0001) in early stage OVCA than the stroma of normal ovaries. The frequency of IgY-containing cells increased further (*P* < 0.0001) in the stroma of the ovaries with tumor at late stages. (b) Although influx of IgY-containing cells increased in the stroma during OVCA progression, infiltration of IgY-containing cells into the tumor did not increase significantly. Bars with different letters indicate significant differences in IgY-containing cells among hens of different pathological groups.

Similar to CD8+ T cells, IgY-containing cells were also localized in the stroma (normal ovaries or ovaries with tumor) as well as in the theca of stromal follicles in normal hens and in the nests of the tumor (intratumoral infiltration) in OVCA hens at early and late stages (Figures 5(a)–5(c)). The mean population of IgY-containing cells in the tumor stroma was significantly (*P* < 0.0001) high in OVCA hens at early stage (mean ± SEM = 21 ± 2.35 cells in 20,000 *μ*m^2^ area of the tissue) compared with the stroma of the normal hens (mean ± SEM = 7 ± 1.10 cells in 20,000 *μ*m^2^ area of the tissue) and increased further in hens with late stage OVCA (mean ± SEM = 35 ± 2 cells in 20,000 *μ*m^2^ area of the tissue) (Figure 6(a)). Although the frequency of stromal IgY-containing cells increased as the OVCA progressed to late stages, the intratumoral influx of IgY-containing cells was not significantly (*P* < 0.10) different between the early (mean ± SEM = 8 ± 1.10 cells in 20,000 *μ*m^2^ area of the tissue) and late stage (mean ± SEM = 9 ± 1.07 cells in 20,000 *μ*m^2^ area of the tissue) OVCA (Figure 6(b)).

### 3.4. Expression of Immune Responsive Gene 1 (IRG1) Protein

**Figure 7:**
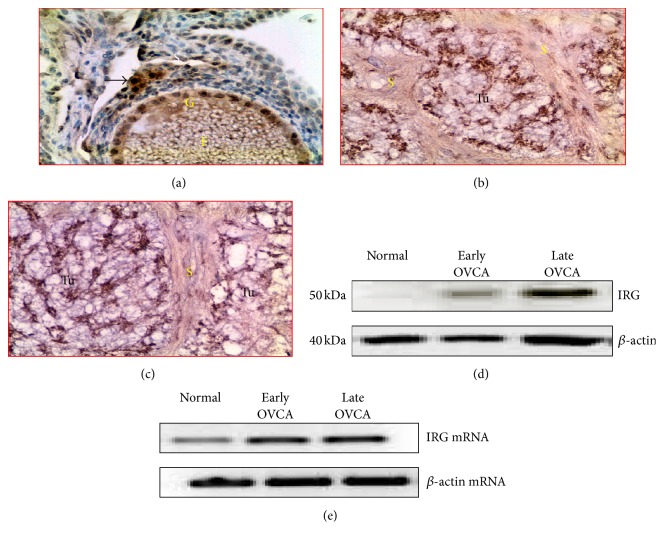
Immunohistochemical detection of IRG1 expression during OVCA development and progression in hens. (a) Normal ovary. Few stroma and granulosa cells of the follicle expressed IRG1. (b-c) Sections of ovarian tumors at early and late stages. Compared with normal ovaries, the tumor expressed IRG1 intensely. (d) Western blotting showed a strong band of approximately 50 kDa in OVCA at early and late stages while it was very weak for normal hens. (e) Semiquantitative PCR showed strong amplification for IRG1 mRNA in tumors compared with normal ovaries. F: follicle; G: granulosa layer of the follicle; S: stroma; Tu: tumor. Magnification: 40x.

Malignant cells in ovaries with tumor showed strong staining for IRG1; however, IRG1 expression was very occasional and weak in the stroma of normal ovaries (Figures 7(a)–7(c)). Immunoblotting for IRG1 expression by homogenates from whole normal or malignant ovaries confirmed the immunohistochemical expression of IRG1. Immunoblotting detected a band of 50 kDa for IRG1 in all groups (Figure 7(d)). As observed in immunohistochemistry, stronger immunoreactive bands for IRG1 were observed for the homogenates of ovaries with tumor than for normal ovaries. Similarly, compared with the normal hens, strong amplification of signals for IRG1 mRNA expression was observed in the ovarian extracts from hens with early and late stages of OVCA (Figure 7(e)) as observed by the immunoreactivities in immunohistochemistry and immunoblotting.

**Figure 8:**
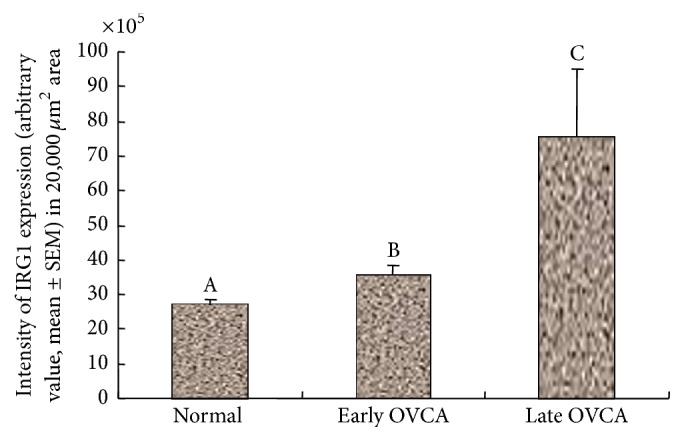
IRG1 expression in normal ovaries and ovarian tumors at early and late stages. Intensities of IRG1 are expressed as arbitrary values (mean ± SEM) in 20,000 *μ*m^2^ area of the tissue. Compared with the normal, the intensity of IRG1 expression was significantly greater in hens with early stage OVCA (*P* < 0.01) and increased further in hens with late stage OVCA (*P* < 0.05). Significant differences were not observed in the intensity of IRG1 expression among hens with serous, endometrioid, and mucinous OVCA at early and late stages. Bars with different letters indicate significant differences in IRG1 expression.

Compared with normal ovaries (mean ± SEM = 27 × 10^5^ ± 1.60 × 10^5^ in 20,000 *μ*m^2^ area), the mean intensity of IRG1 expression in early stage OVCA was significantly high (mean ± SEM = 35 × 10^5^ ± 3.1 × 10^5^ in 20,000 *μ*m^2^ area, *P* < 0.01) and increased further in late stage OVCA (mean ± SEM = 75 × 10^5^ ± 19.50 × 10^5^ in 20,000 *μ*m^2^ area, *P* < 0.02) (Figure 8). Significant differences were not observed in the expression of IRG1 among different histological types including serous, endometrioid, and mucinous OVCA at early and late stages.

### 3.5. Expression of DR6

**Figure 9:**
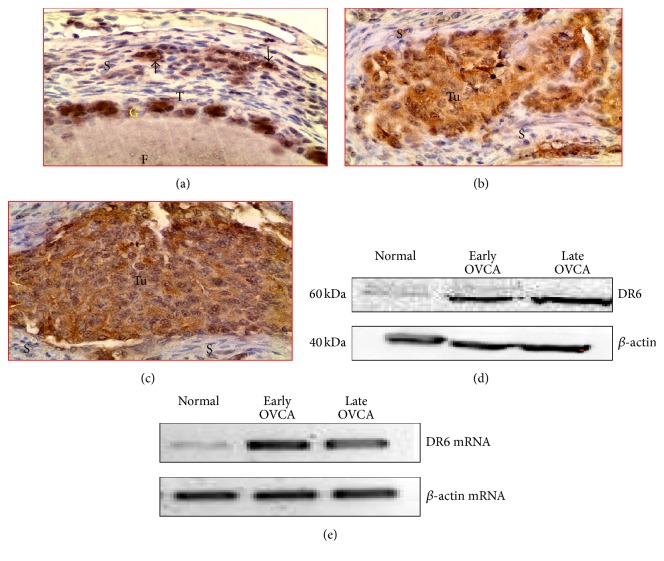
DR6 expression in normal or tumor ovaries. (a) Normal ovary. Few stromal and granulosa cells of the follicle expressed DR6. (b-c) Ovarian tumors at early and late stages showing strong expression of DR6. (d) Western blotting detected the DR6 protein of 50–60 kDa in normal or malignant ovaries. Compared with the normal, stronger bands for DR6 proteins were observed in early and late stages of OVCA. (e) Semiquantitative mRNA expression assays showed strong amplification for DR6 in tumor compared with normal ovaries. F: follicle; G and T: granulosa and theca layers of the follicle, respectively; S: stroma; Tu: tumor. Magnification: 40x.

Intense expression of DR6 was detected in the malignant cells in ovaries with tumor at early and late stage OVCA while few OSE cells in normal ovaries showed weak to moderate staining for DR6 (Figures 9(a)–9(c)). Immunohistochemical expression of DR6 by the normal ovaries and ovarian tumors was confirmed by immunoblotting using homogenates from whole normal or malignant ovaries. Similar immunoreactions with a band size of 60 kDa were detected in the tissue homogenates (Figure 9(d)). As observed in the immunohistochemical study, compared with normal ovaries, intense immunoreactions for DR6 were detected in malignant ovaries. Compared with the normal hens, strong amplification of signal for DR6 mRNA (Figure 9(e)) was observed in the ovarian extracts from hens with early and late stages of OVCA as observed for immunoreactivities in immunohistochemistry and immunoblotting.

**Figure 10:**
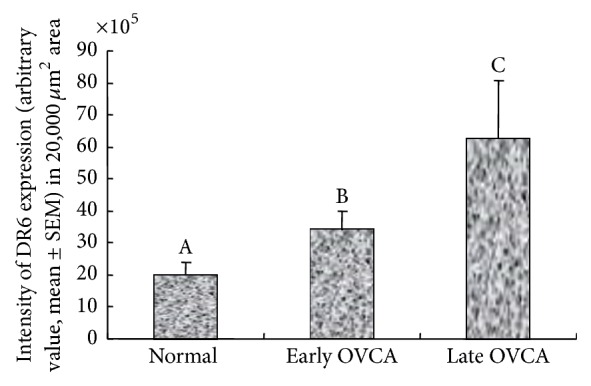
Changes in DR6 expression during ovarian cancer (OVCA) development and progression. Compared with normal ovaries, the intensities of the DR6 expression (arbitrary values expressed as mean ± SEM in 20,000 *μ*m^2^ area of the tissue) were significantly higher in hens with early stage OVCA (*P* < 0.01) and increased further in hens with late stage OVCA (*P* < 0.05). However, significant differences were not observed in the intensity of DR6 expression among hens with serous, endometrioid, and mucinous OVCA at late stages. Bars with different letters indicate significant differences in DR6 expression.

The mean intensity of DR6 expression in early stage OVCA (mean ± SEM = 34 × 10^5^ ± 5.5 × 10^5^ in 20,000 *μ*m^2^ area) was significantly high (*P* < 0.01) as compared with normal ovaries (mean ± SEM = 20 × 10^5^ ± 4.1 × 10^5^ in 20,000 *μ*m^2^ area). The mean intensity of DR6 expression increased further (mean ± SEM = 62 × 10^5^ ± 18.4 × 10^5^ in 20,000 *μ*m^2^ area) in late stage OVCA (*P* < 0.05) (Figure 10). Significant differences in DR6 staining intensities were not observed among 3 different histological subtypes of OVCA at early and late stages.

## 4. Discussion

This is the first report on the changes in the frequency of cell mediated cytotoxic and humoral immune function relative to the expression of tumor-induced immunosuppressive factors including IRG1 and DR6 during OVCA development and progression in the laying hen model of spontaneous OVCA. This study showed the following. (1) Influx of immune cells (including CD8+ T cells and IgY-containing cells) into the tumor stroma increased in response to tumor development; however, infiltration of these cells into the tumor nests during OVCA progression did not increase significantly. (2) Tumor development and progression were associated with increased expression of tumor-associated stress factor DR6 and tumor-induced immunosuppressor IRG1.

Tumor development and progression are associated with elicitation of antitumor immune responses including the activation of both cytotoxic T lymphocytes and antibody mediated immune functions [32]. However, these spontaneous antitumor immune responses appeared to be inefficient as the tumor continues to grow and natural rejection of solid tumors is rare [4, 33]. The mechanism(s) of suppression of antitumor immune function involved in the development and progression of tumor is not known. Emerging information suggests that the tumor escapes antitumor immune functions by inducing immunosuppressive factors [4]. This study showed that, compared with age-matched normal ovaries, the frequency of CD8+ T cells and IgY-containing cells in the tumor stroma increased significantly at early stage and increased further at late stages. Thus, these results suggest that influx of CD8+ T cells and IgY-containing cells into the tumor vicinity increases in response to OVCA development and progression. In contrast, significant changes were not observed in the frequency of intratumoral CD8+ T cells and IgY-containing cells between the OVCA at early and late stages. These results indicate that although influx of CD8+ T cells and IgY-containing cells increased into the tumor stroma during tumor progression, the rate of intratumoral infiltration of CD8+ T and IgY-containing cells was not increased. Thus, lower rate of intratumoral infiltration of immune cells may be a reason allowing the tumor to progress to late stages. However, factors associated with low rate of intratumoral CD8+ T cells are not known. It is possible that tumor-induced factor(s) may be involved in the inhibition of immune cell infiltration into the tumor.

In this study, strong expression for immunoresponsive gene 1 (IRG1) protein was observed in the malignant cells at early and late stages of OVCA. IRG1 is highly expressed under proinflammatory conditions [33, 34] in both mammals and avian species [35]. IRG1 is also highly expressed by the uterus during implantation of fetuses which is also accompanied with high levels of proinflammatory cytokine production [36]. All these reports suggest that IRG1 plays critical roles to suppress antitumor immunity and allow the tumor to progress. Similarly, IRG1 has also been reported to induce tolerance during fetal implantation in the uterus [37]. Thus, based on the observations of the present study, it is possible that increased expression of IRG1 may be associated with the immune tolerance and decreased rate of intratumoral infiltration of immune cells during OVCA development and progression. However, the mechanism of OVCA-induced increased expression of IRG1 in the inhibition of intratumoral infiltration of CD8+ T cells and IgY-containing cells is not known. Emerging information suggests that enhanced IRG1 is associated with the increased generation of reactive oxygen species and hypoxia [38].

Malignant transformation in the ovary was shown to be associated with increased secretion of chemoattractant cytokines resulting in the influx of immune cells into the tumor microenvironment. Increased immune cells in the tumor vicinity increase the demand for oxygen leading to a hypoxic condition resulting in the production of reactive oxygen species. DR6 expression has been shown to increase in association with hypoxia and this study showed increased expression of DR6 by the tumor cells at early and late stages of OVCA. Furthermore, increase in expression of IRG1 was positively associated with enhanced DR6 expression during OVCA development and progression. Thus, it is possible that increase in DR6 expression during OVCA development and progression may lead to the increased expression of IRG1. Increased IRG1 expression by ovarian malignant cells may be a factor for the decreased infiltration of CD8+ T cells and IgY-containing cells into the tumor.

The results of the present study offer several translational aspects with respect to the prevention of OVCA development and progression as well as recurrence. This study, for the first time, revealed an association of tumor-induced IRG1 expression and CD8+ T cell infiltration of ovarian tumors. Thus, targeting tumor-associated hypoxia as well as DR6 expression may reduce IRG1 expression and thus may enhance infiltration of immune cells into the tumor. Because immunotherapy is safer than other conventional chemotherapeutics as well as radiotherapies, this animal model of spontaneous OVCA may be used to develop targeted immunotherapies against OVCA. As this model resembles human OVCA in histopathology and tumor progression, results from this study may be translated to the clinical study with minor modification.

## 5. Conclusions

Taken together, the results of the current study showed that ovarian tumor progression is associated with the decreased rate of intratumoral infiltration of CD8+ T cells and IgY-containing cells as well as increased expression of IRG1. Increased expression of IRG1 was associated with the enhanced expression of DR6 during OVCA development and progression.
